# Evaluation of surgical treatment’s impact on overall survival in elderly patients with stage IV non-small cell lung cancer using the propensity score matching method

**DOI:** 10.1097/MD.0000000000047224

**Published:** 2026-01-23

**Authors:** Feiyang Li, Fang Li

**Affiliations:** a Ward 2, Department of Medical Oncology, Lixin People’s Hospital of Bozhou City, Bozhou, Anhui Province, China; b Ward 1, Department of Medical Oncology, Affiliated Hospital of Qinghai University, Xining, Qinghai Province, China.

**Keywords:** non-small cell lung cancer, overall survival, propensity score matching, SEER database, surgical treatment

## Abstract

A retrospective analysis was conducted on patients aged 60 and above with stage IV non-small cell lung cancer (NSCLC), in order to provide more evidence regarding the extension of the overall survival (OS) period of this patient group through surgical treatment. Patient data from the surveillance, epidemiology, and end results database between 2010 and 2015 were screened to identify cases with pathologically confirmed diagnoses of NSCLC. The inclusion criteria encompassed patients aged 60 years and above diagnosed with stage IV disease, as defined by the American Joint Committee on Cancer (AJCC) staging system. The eligible patients were categorized into surgical and non-surgical cohorts. A balanced comparison between the 2 cohorts was achieved through propensity score matching (PSM). Matching variables encompassed age, sex, race, marital status, tumor location, AJCC T stage, AJCC N stage, receipt of radiotherapy or chemotherapy, and the presence of distant metastases (bone, brain, lung, and liver). Kaplan–Meier analysis and multivariable Cox regression analysis were performed on the matched data to assess the effect of surgery on OS. To elucidate the potential benefits of surgical treatment across distinct patient populations, a detailed subgroup analysis was conducted including baseline variables between the surgical and non-surgical groups to accurately identify subgroups that benefit from surgical interventions. A total of 14,495 patients were included in the study, with 504 undergoing surgical intervention and 13,991 receiving non-surgical management. Following PSM, 1008 patients were successfully matched to establish balanced comparison groups. Multivariate Cox regression analysis revealed that surgical intervention was associated with a 54% reduction in mortality risk (hazard ratio = 0.46; 95% CI: 0.40–0.52; *P* <.001). Kaplan–Meier survival analysis demonstrated a significant advantage for the surgical group compared to the non-surgical group, with median OS of 21 months versus 7 months, respectively (*P* <.001). Subgroup analysis indicated that patients who did not undergo chemotherapy and those without lung metastases experienced greater survival benefit from surgical intervention. Unlike previous studies, this research specifically targets elderly patients diagnosed with stage IV NSCLC. The findings suggest that surgical intervention may enhance the prognosis in this cohort. Nevertheless, the decision to proceed with surgery must be based on a thorough assessment of the patient’s clinical condition and comorbidities.

## 1. Introduction

In 2022, lung malignancies were the most prevalent malignant tumors, accounting for 2.5 million new cases and 1.796 million deaths worldwide. Projections indicate that by 2050, the number of new lung cancer cases in the United States will reach 3.8 million, while the number of deaths is expected to rise to 3.2 million. Currently and in the foreseeable future, lung cancer is expected to remain a significant public health issue in the United States.^[1,2]^ In the United States, approximately 84% of patients with lung malignancies are diagnosed with non-small cell lung cancer (NSCLC), making it the predominant pathological subtype of lung malignancies. Owing to the absence of specific symptoms in the early stages of NSCLC, around 44% of patients are diagnosed with distant metastasis, corresponding to Stage IV according to the American Joint Committee on Cancer (AJCC) staging system. The prognosis for this group of patients is particularly poor.^[3]^ For patients with distant metastasis, systemic therapies, including chemotherapy, targeted therapy, and immunotherapy, are the mainstay of treatment. Surgical treatment is not recommended for this group of patients. Studies have shown that during the chemotherapy era, the median overall survival (OS) of patients with Stage IV NSCLC was approximately 11 months. In recent years, with the development of molecular targeted therapies and immune checkpoint inhibitors (ICIs), the OS of these patients has improved modestly; however, survival duration remains limited.^[4–7]^

In recent years, with the growing understanding and acceptance of the concept of oligometastasis, advancements in treatment methods, and the emphasis on personalized therapy, the role of surgical treatment in Stage IV NSCLC has evolved significantly. An increasing number of experts believe that a subset of patients in this group can benefit from surgery. Takeshi Hanagiri et al performed a retrospective analysis on 36 Stage IV NSCLC patients undergoing primary tumor resection and found that their 5-year OS rate reached 26.8%. In contrast, the historical survival rate for Stage IV NSCLC patients is below 10%.^[8,9]^ Another retrospective analysis also showed that primary tumor resection significantly enhances survival outcomes for patients with Stage IV NSCLC. Among patients who underwent complete resection of the primary tumor, the 5-year survival rate was 29.0%. The study further found that patients with non-squamous NSCLC experienced greater survival benefits from surgery.^[10]^ The results of these 2 studies are similar, both suggesting that surgical treatment is a viable option for Stage IV NSCLC patients. However, careful patient selection is essential, as both studies focused on patients with oligometastatic disease rather than those with widespread metastasis.

However, these studies did not specifically analyze the elderly patient population, representing a notable limitation. The lack of data for this group limits the generalizability and specific clinical applicability of the study findings. Currently, the median age at diagnosis for lung malignancies is 70 years, and approximately 70% of NSCLC patients are aged 65 years or older. The elderly population has become the main subgroup of NSCLC patients, and as the population continues to age, this proportion is projected to increase further.^[11–13]^ Elderly patients often experience a decline in lean body mass, strength, endurance, balance, and mobility, resulting in significantly higher mortality rates at 30 days, 90 days, and 1 year post-surgery.^[14]^ Therefore, whether elderly patients with Stage IV NSCLC can benefit from surgery remains a topic of debate and investigation. Motivated by this uncertainty, we conducted a study on surgical treatment for elderly patients with Stage IV NSCLC. This study aims to offer critical insights into the surgical treatment of elderly patients diagnosed with stage IV NSCLC, thereby contributing to the formulation of personalized treatment strategies. The results of this study are anticipated to provide valuable references and insights for the design of phase III clinical trials, particularly with regard to trial protocol development and influencing factors.

## 2. Materials and methods

### 2.1. Data sources and patient selection

**Figure 1.**
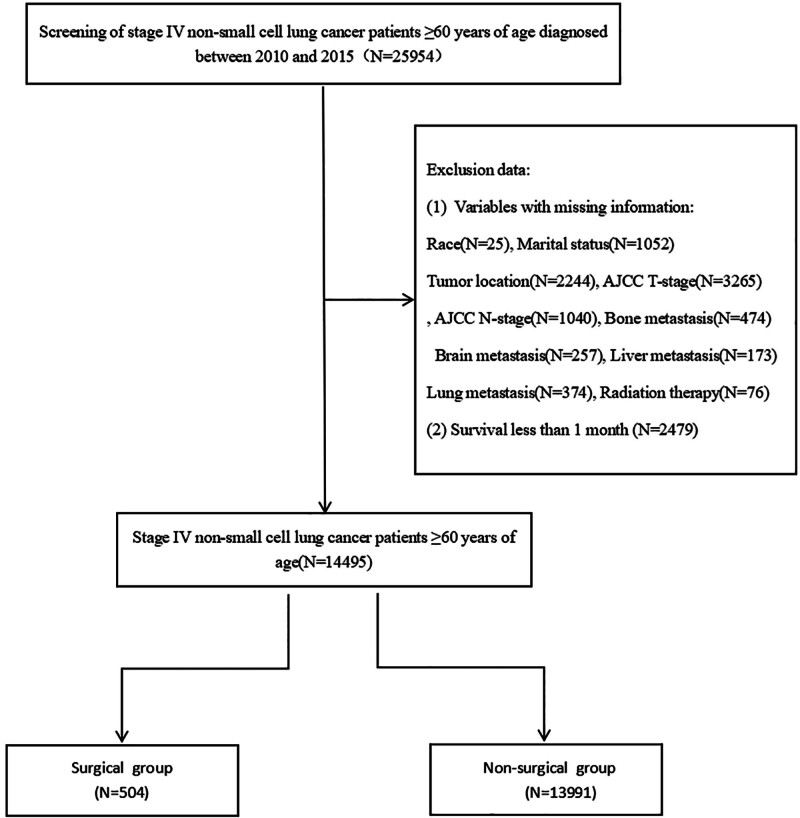
Screening patient flowchart.

We obtained data on lung malignancies using SEER*Stat 8.4.3, containing data on tumor staging, clinicopathological features, and survival outcomes. The surveillance, epidemiology, and end results (SEER) database is a public research resource established by the National Cancer Institute of the United States, covering approximately 30% of the population in North America. The vast amount of data and diversity in the SEER database enhance the reliability and universality of our research results.^[15]^ Before initiating this study, we submitted a data use agreement to the SEER program and received official approval for database access (approval number: 14038-Nov2021). SEER is a publicly accessible research database, and the personal information within the database has been de-identified. Therefore, this study did not require ethical approval or informed consent. The study was conducted in accordance with the Declaration of Helsinki (2013 revision). We screened the data according to the established inclusion and exclusion criteria. The inclusion criteria are as follows: patients diagnosed with Stage IV based on AJCC 7th edition staging; diagnosed between 2010 and 2015; and age ≥ 60 years at diagnosis. Exclusion criteria included: patients with missing key information; patients with ICD-O-3 pathologic diagnoses including 8002/3 (malignant tumor, small cell type), 8041/3 (small cell carcinoma, NOS), 8043/3 (small cell carcinoma, fusiform cell), 8044/3 (small cell carcinoma, intermediate cell), and 8045/3 (combined small cell carcinoma); and patients with a survival duration of <1 months. The specific screening process is shown in Figure 1.

### 2.2. Data collection

Data collected included age at diagnosis (60–71 years, 72–80 years, and ≥81 years); sex (male or female); race (white, black, or other races); marital status (divorced, married, or single); tumor location (left lung or right lung); T-stage (T1, T2, T3, T4); N-stage (N0-1, N2, N3); radiation therapy (yes or no); chemotherapy (yes or no); surgical treatment (yes or no); sites of distant metastasis (bone, brain, liver, lung); OS time; and survival status. For age stratification, we primarily utilized X-tile software (version 3.6.1). We applied the enumeration method to divide age into 3 quartile groups and determined the optimal cutoff values.

### 2.3. Statistical analysis

This study performed a descriptive statistical analysis on the patient data. Categorical variables were expressed as frequencies and percentages. A χ^2^ test was also applied to compare the demographic characteristics and tumor features between the surgical treatment group and the non-surgical treatment group; To address baseline data imbalance and control for potential confounding factors, we employed the propensity score matching (PSM) method. We calculated the propensity score (PS) for each patient and performed a 1:1 matching using the MatchIt package in R, setting the truncation width to 0.25 times the standard deviation of the PS. Subsequently, we assessed the balance of baseline characteristics between groups after PSM using the standardized mean difference (SMD), with an SMD <0.1 considered acceptable; Kaplan–Meier curves were used to illustrate survival rates at various follow-up time points, and the log-rank test was applied to compare survival differences between the 2 groups; We constructed a multivariable Cox proportional hazards regression model that incorporated variables such as age, gender, race, marital status, tumor location, AJCC T stage, AJCC N stage, radiotherapy, chemotherapy, and distant metastasis. We calculated the hazard ratio (HR) and 95% confidence interval (95% confidence interval [CI]) to evaluate the effect of surgical treatment; Finally, we performed a subgroup analysis based on variables such as age, gender, race, marital status, tumor location, AJCC T stage, AJCC N stage, radiotherapy, chemotherapy, and distant metastasis to calculate the HR values and their 95% confidence intervals for each subgroup. All statistical analyses were performed using R software (version 4.3.1), with a *P*-value threshold of <.05 considered statistically significant.

## 3. Results

### 3.1. Baseline information

In the surgical group, the proportion of patients aged 60 to 71 years was higher than in the non-surgical group (56.94% vs 45.38%), whereas the proportion of patients aged 81+ years was significantly lower in the surgical group than in the non-surgical group (8.53% vs 21.29%). This suggests that age is a critical factor influencing the choice of surgical treatment. Regarding marital status, the proportions of married, single, and divorced patients in the surgical group were 62.7%, 22.42%, and 14.88%, respectively. In the non-surgical group, the corresponding proportions were 53.91%, 24.79%, and 21.30%, respectively. In both groups, the proportion of married patients was >50%. This may indicate that married patients are more likely to receive family support when opting for surgery. In terms of AJCC N stage, the proportion of N0-1 patients was significantly higher in the surgical group than in the non-surgical group (69.05% vs 35.89%). Fewer regional lymph node metastases seem to be a key factor influencing surgical decisions in Stage IV NSCLC patients. For distant metastasis, the proportions of bone, brain, lung, and liver metastases were all lower in the surgical group than in the non-surgical group, with liver metastases observed in 5.36% of the surgical group compared to 16.31% of the non-surgical group. Additionally, the most common site of metastasis in the surgical group was the lung (26.79%), while in the non-surgical group, it was the bone (38.95%). This may be because lung metastases are relatively localized and more amenable to resection. The differences in baseline characteristics between the 2 groups were statistically significant (*P* <.001) (Table 1).

**Table 1:** Baseline characteristics of patients before PSM.

Variable	Total (n = 14,495)	No (n = 13,991)	Yes (n = 504)	Statistic	*P*
Age, n (%)					
60–71 yr	6636 (45.78)	6349 (45.38)	287 (56.94)	χ^2^ = 52.416	*<*.001
72–80 yr	4837 (33.37)	4663 (33.33)	174 (34.52)		
81+ yr	3022 (20.85)	2979 (21.29)	43 (8.53)		
Sex, n (%)					
Female	6697 (46.2)	6442 (46.04)	255 (50.60)	χ^2^ = 4.054	.044
Male	7798 (53.8)	7549 (53.96)	249 (49.40)		
Race, n (%)					
Black	1191 (8.22)	1167 (8.34)	24 (4.76)	χ^2^ = 13.369	.001
Other*	1812 (12.5)	1763 (12.60)	49 (9.72)		
White	11,492 (79.28)	11,061 (79.06)	431 (85.52)		
Marital status, n (%)					
Married	7859 (54.22)	7543 (53.91)	316 (62.70)	χ^2^ = 17.537	*<*.001
Single	3581 (24.71)	3468 (24.79)	113 (22.42)		
Widowed	3055 (21.08)	2980 (21.30)	75 (14.88)		
Laterality, n (%)					
Left	6125 (42.26)	5903 (42.19)	222 (44.05)	χ^2^ = 0.687	.407
Right	8370 (57.74)	8088 (57.81)	282 (55.95)		
AJCC T, n (%)					
T1	1871 (12.91)	1796 (12.84)	75 (14.88)	χ^2^ = 3.932	.269
T2	4136 (28.53)	3983 (28.47)	153 (30.36)		
T3	3914 (27)	3781 (27.02)	133 (26.39)		
T4	4574 (31.56)	4431 (31.67)	143 (28.37)		
AJCC N, n (%)					
N0-1	5370 (37.05)	5022 (35.89)	348 (69.05)	χ^2^ = 236.077	<.001
N2	6312 (43.55)	6183 (44.19)	129 (25.60)		
N3	2813 (19.41)	2786 (19.91)	27 (5.36)		
Radiation, n (%)					
No	7882 (54.38)	7555 (54.00)	327 (64.88)	χ^2^ = 23.221	<.001
Yes	6613 (45.62)	6436 (46.00)	177 (35.12)		
Chemotherapy, n (%)					
No	6280 (43.33)	6060 (43.31)	220 (43.65)	χ^2^ = 0.023	.881
Yes	8215 (56.67)	7931 (56.69)	284 (56.35)		
Bone metastasis, n (%)					
No	8967 (61.86)	8542 (61.05)	425 (84.33)	χ^2^ = 111.672	<.001
Yes	5528 (38.14)	5449 (38.95)	79 (15.67)		
Brain metastasis, n (%)					
No	10,907 (75.25)	10,524 (75.22)	383 (75.99)	χ^2^ = 0.156	.693
Yes	3588 (24.75)	3467 (24.78)	121 (24.01)		
Liver metastasis, n (%)					
No	12,186 (84.07)	11,709 (83.69)	477 (94.64)	χ^2^ = 43.582	<.001
Yes	2309 (15.93)	2282 (16.31)	27 (5.36)		
Lung metastasis, n (%)					
No	10,217 (70.49)	9848 (70.39)	369 (73.21)	χ^2^ = 1.868	.172
Yes	4278 (29.51)	4143 (29.61)	135 (26.79)		

χ^2^ = Chi-square test, AJCC = American Joint Committee on Cancer, PSM = propensity score matching.

* American Indian/AK Native, Asian/Pacific Islander.

### 3.2. PSM between the 2 groups

**Figure 2.**
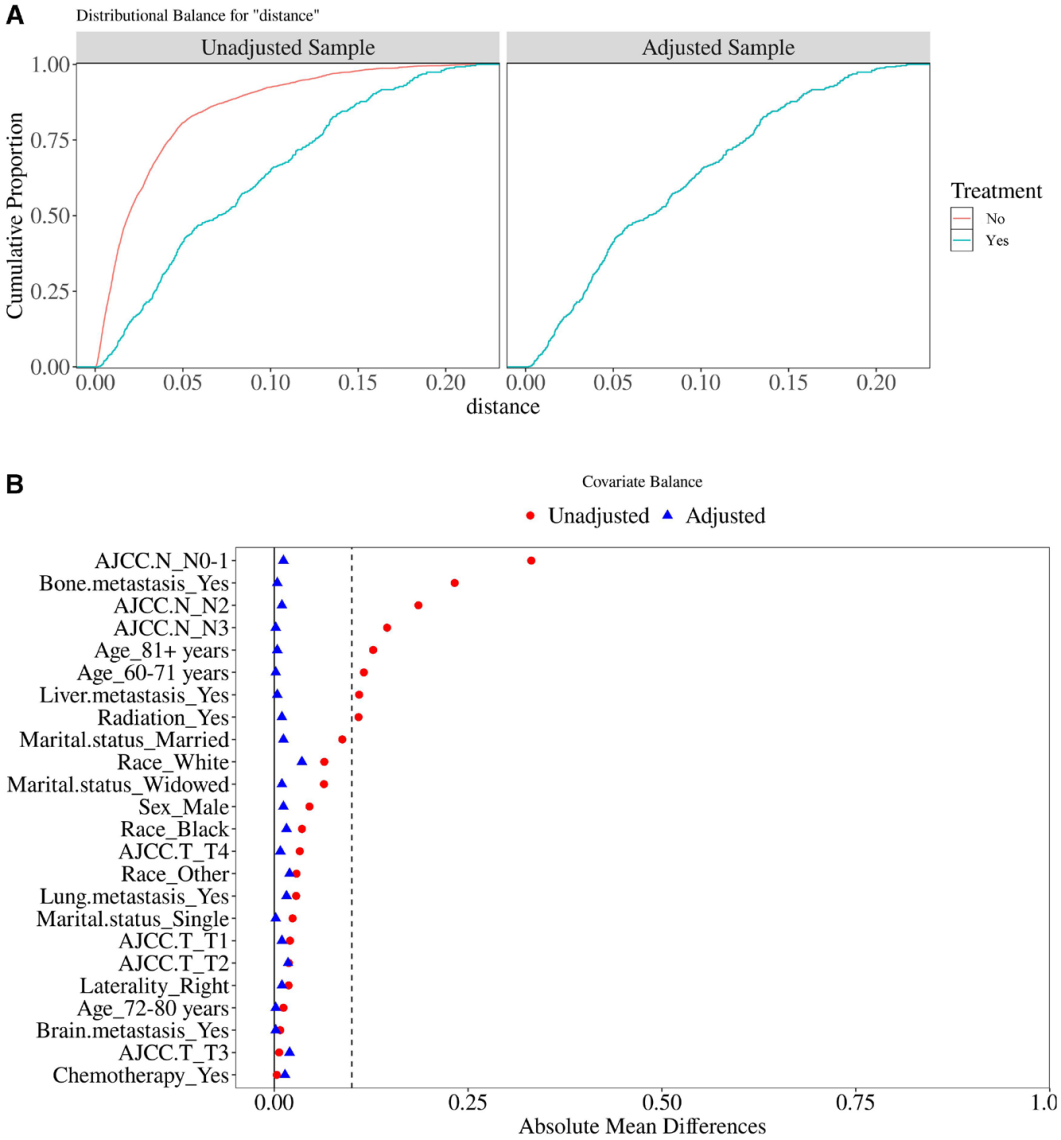
(A) Shows the cumulative proportions of the 2 groups; (B) Shows the absolute standardized mean of the 2 groups (Non-adjusted groups: Pre-matching data; Adjusted groups: Post-matching data).

Baseline imbalances were observed between the 2 groups, potentially introducing bias into the results. To reduce this bias and account for potential confounding factors, we applied PSM. PSM integrates multiple covariates into a PS, and each treated individual is matched with one or more untreated individuals who have similar PSs. This approach significantly reduces selection bias and mitigates potential confounding factors in observational studies, and its results can be comparable to those of randomized controlled trials.^[16]^ The PSM method not only addresses the issue of baseline imbalance but also enhances the robustness of our results. Using a logistic regression model, PS were calculated based on covariates including age, gender, race, marital status, tumor location, AJCC T stage, AJCC N stage, radiotherapy, chemotherapy, and distant metastases (bone, brain, lung, liver). Matching was performed using the MatchIt package in R in a 1:1 ratio between patients undergoing surgical treatment and those who did not. The caliper width was set to 0.25 standard deviations of the PS. The balance of baseline characteristics after PSM was evaluated based on SMD, with values below 0.1 indicating acceptable balance. A total of 1008 patients were successfully matched, resulting in well-balanced baseline characteristics between the 2 groups, with no statistically significant differences (all *P*-values >.05) (Table 2). Figure 2A shows the cumulative proportions for the 2 groups, with nearly identical curves after matching. Figure 2B displays the absolute standardized mean differences, concentrated near zero after matching, confirming the absence of systematic differences (Fig. 2).

**Table 2:** Baseline characteristics of patients after PSM.

Variable	Total (n = 1008)	No (n = 504)	Yes (n = 504)	Statistic	*P*	SMD
Age, n (%)						
60–71 yr	573 (56.85)	286 (56.75)	287 (56.94)	χ^2^ = 0.050	.975	0.004
72–80 yr	347 (34.42)	173 (34.33)	174 (34.52)			0.004
81+ yr	88 (8.73)	45 (8.93)	43 (8.53)			−0.014
Sex, n (%)						
Female	504 (50)	249 (49.40)	255 (50.60)	χ^2^ = 0.143	.705	0.024
Male	504 (50)	255 (50.60)	249 (49.40)			−0.024
Race, n (%)						
Black	40 (3.97)	16 (3.17)	24 (4.76)	χ^2^ = 3.105	.212	0.075
Other*	88 (8.73)	39 (7.74)	49 (9.72)			0.067
White	880 (87.3)	449 (89.09)	431 (85.52)			−0.101
Marital status, n (%)						
Married	638 (63.29)	322 (63.89)	316 (62.70)	χ^2^ = 0.233	.890	−0.025
Single	225 (22.32)	112 (22.22)	113 (22.42)			0.005
Widowed	145 (14.38)	70 (13.89)	75 (14.88)			0.028
Laterality, n (%)						
Left	439 (43.55)	217 (43.06)	222 (44.05)	χ^2^ = 0.101	.751	0.020
Right	569 (56.45)	287 (56.94)	282 (55.95)			−0.020
AJCC T, n (%)						
T1	145 (14.38)	70 (13.89)	75 (14.88)	χ^2^ = 0.863	.834	0.028
T2	297 (29.46)	144 (28.57)	153 (30.36)			0.039
T3	276 (27.38)	143 (28.37)	133 (26.39)			−0.045
T4	290 (28.77)	147 (29.17)	143 (28.37)			−0.018
AJCC N, n (%)						
N0-1	690 (68.45)	342 (67.86)	348 (69.05)	χ^2^ = 0.165	.921	0.026
N2	263 (26.09)	134 (26.59)	129 (25.60)			−0.023
N3	55 (5.46)	28 (5.56)	27 (5.36)			−0.009
Radiation, n (%)						
No	649 (64.38)	322 (63.89)	327 (64.88)	χ^2^ = 0.108	.742	0.021
Yes	359 (35.62)	182 (36.11)	177 (35.12)			−0.021
Chemotherapy, n (%)						
No	447 (44.35)	227 (45.04)	220 (43.65)	χ^2^ = 0.197	.657	−0.028
Yes	561 (55.65)	277 (54.96)	284 (56.35)			0.028
Bone metastasis, n (%)						
No	852 (84.52)	427 (84.72)	425 (84.33)	χ^2^ = 0.030	.862	−0.011
Yes	156 (15.48)	77 (15.28)	79 (15.67)			0.011
Brain metastasis, n (%)						
No	765 (75.89)	382 (75.79)	383 (75.99)	χ^2^ = 0.005	.941	0.005
Yes	243 (24.11)	122 (24.21)	121 (24.01)			−0.005
Liver metastasis, n (%)						
No	952 (94.44)	475 (94.25)	477 (94.64)	χ^2^ = 0.076	.783	0.018
Yes	56 (5.56)	29 (5.75)	27 (5.36)			−0.018
Lung metastasis, n (%)						
No	746 (74.01)	377 (74.80)	369 (73.21)	χ^2^ = 0.330	.566	−0.036
Yes	262 (25.99)	127 (25.20)	135 (26.79)			0.036

χ^2^ = Chi-square test, AJCC = American Joint Committee on Cancer, PSM = propensity score matching, SMD = standardized mean differences.

* American Indian/AK Native, Asian/Pacific Islander.

### 3.3. Kaplan–Meier survival analysis

**Figure 3.**
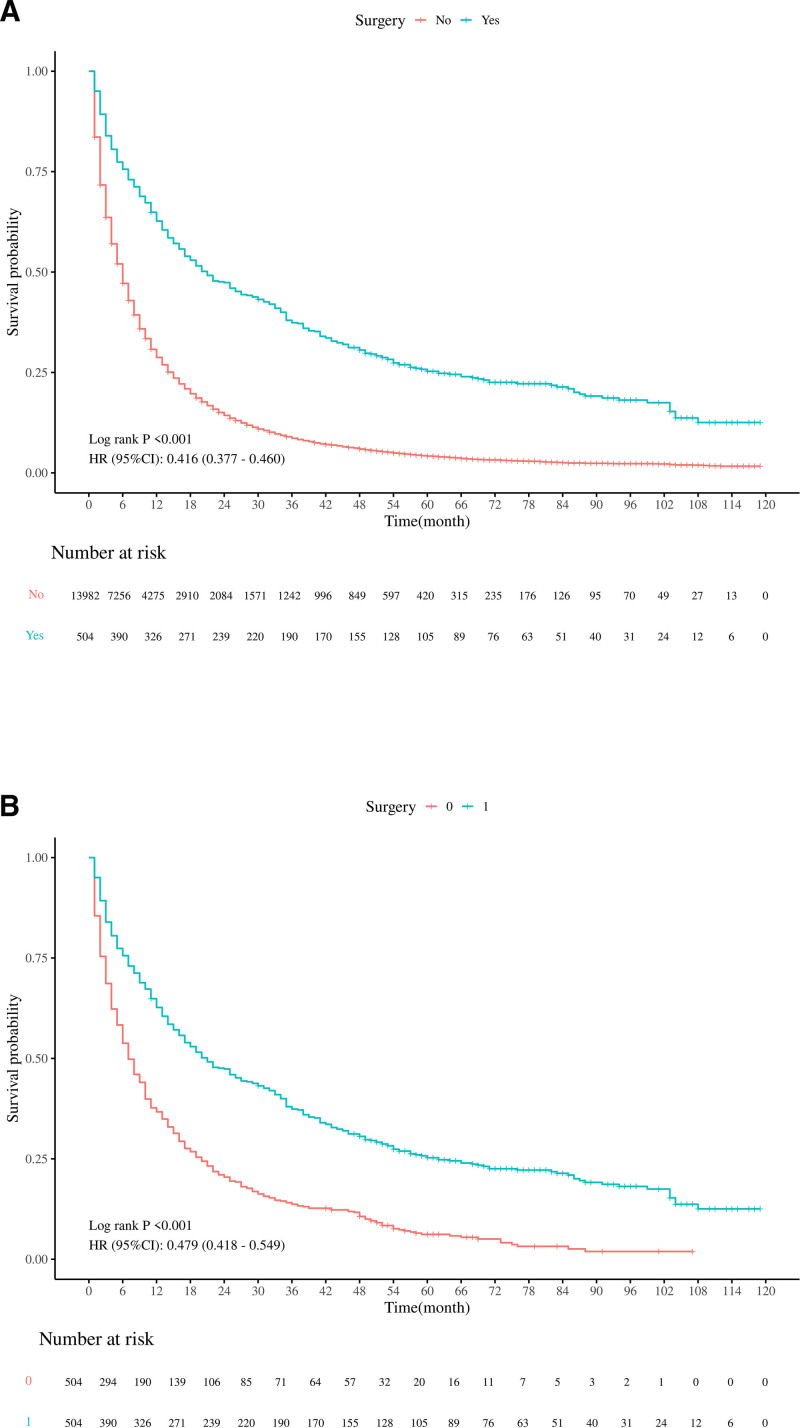
(A) Shows the Kaplan–Meier survival curves for OS time in the 2 groups before PSM; (B) Shows the Kaplan–Meier survival curves for OS time in the 2 groups after PSM. OS = overall survival, PSM = propensity score matching.

Using the pre-matching dataset, Kaplan–Meier curve analysis demonstrated that the OS in the surgical treatment group was superior to that of the non-surgical treatment group, with patients in the surgical group experiencing a 58.4% reduction in the risk of death (HR = 0.416, 95% CI: 0.377–0.460) (Fig. 3A). However, the results’ reliability was limited by baseline imbalances between the 2 groups. To address this limitation, PSM was performed to balance baseline characteristics. Kaplan–Meier curve analysis of the matched dataset confirmed that the surgical treatment group maintained superior OS compared to the non-surgical group. The median OS was 21 months versus 7 months (*P* <.001), with the surgical group showing a 52.1% reduced risk of death (HR = 0.479, 95% CI: 0.418–0.549) (Fig. 3B). The Log-rank test demonstrated that the difference between the 2 groups was statistically significant (*P* <.001). Moreover, the 1-year, 3-year, and 5-year survival rates for the surgical group were 64.7%, 37.7%, and 20.0%, respectively, while those for the non-surgical group were 37.7%, 14.1%, and 4.0%.

### 3.4. Unifactorial and multifactorial Cox analysis

Univariate Cox regression analysis of the matched dataset identified age, gender, AJCC N stage, surgical treatment, chemotherapy, brain metastasis, liver metastasis, and lung metastasis as potential factors influencing OS (*P* <.1). These factors were subsequently included in a multivariate Cox regression model, which identified them as independent prognostic factors for OS (*P* <.005). Multivariate Cox analysis showed that patients undergoing surgical treatment had a 54% lower risk of death compared to those without surgery (HR = 0.46, 95% CI: 0.40–0.52, *P* <.001) (Table 3).

**Table 3:** Univariate and multivariate Cox analyses of the post-PSM dataset.

Variables	Univariate analysis			Multivariate analysis		
	*P*	HR	95% CI	*P*	HR	95% CI
Age						
60–71 yr	Reference			Reference		
72–80 yr	.071	1.14	0.99–1.32	.018	1.19	1.03–1.38
81+ yr	<.001	1.58	1.25–1.99	<.001	1.58	1.25–2.00
Sex						
Female	Reference			Reference		
Male	<.001	1.34	1.17–1.53	<.001	1.28	1.12–1.46
Race						
Black	Reference			–	–	–
Other*	.246	0.79	0.53–1.18	–	–	–
White	.465	0.88	0.63–1.23	–	–	–
Marital status						
Married	Reference			–	–	–
Single	.317	1.09	0.92–1.28	–	–	–
Widowed	.199	1.13	0.94–1.37	–	–	–
Laterality						
Left	Reference			–	–	–
Right	.149	1.10	0.97–1.26	–	–	–
AJCC T						
T1	Reference			–	–	–
T2	.197	1.15	0.93–1.43	–	–	–
T3	.516	1.07	0.86–1.34	–	–	–
T4	.274	1.13	0.91–1.40	–	–	–
AJCC N						
N0-1	Reference			Reference		
N2	<.001	1.32	1.14–1.54	<.001	1.43	1.22–1.68
N3	<.001	1.67	1.26–2.21	<.001	1.80	1.35–2.41
Surgery						
No	Reference			Reference		
Yes	<.001	0.49	0.43–0.56	<.001	0.46	0.40–0.52
Radiation						
No	Reference			–	–	–
Yes	.172	1.10	0.96–1.26	–	–	–
Chemotherapy						
No	Reference			Reference		
Yes	<.001	0.76	0.66–0.87	<.001	0.63	0.54–0.72
Bone metastasis						
No	Reference			–	–	–
Yes	.175	1.14	0.95–1.36	–	–	–
Brain metastasis						
No	Reference			Reference		
Yes	.040	1.17	1.01–1.37	.006	1.26	1.07–1.48
Liver metastasis						
No	Reference			Reference		
Yes	<.001	1.73	1.31–2.29	<.001	1.89	1.43–2.51
Lung metastasis						
No	Reference			Reference		
Yes	.025	0.84	0.72–0.98	.002	0.78	0.66–0.91

χ^2^ = Chi-square test, AJCC = American Joint Committee on Cancer, CI = confidence interval, HR = hazard ratio, PSM = propensity score matching.

* American Indian/AK Native, Asian/Pacific Islander.

### 3.5. Subgroup analysis

**Figure 4.**
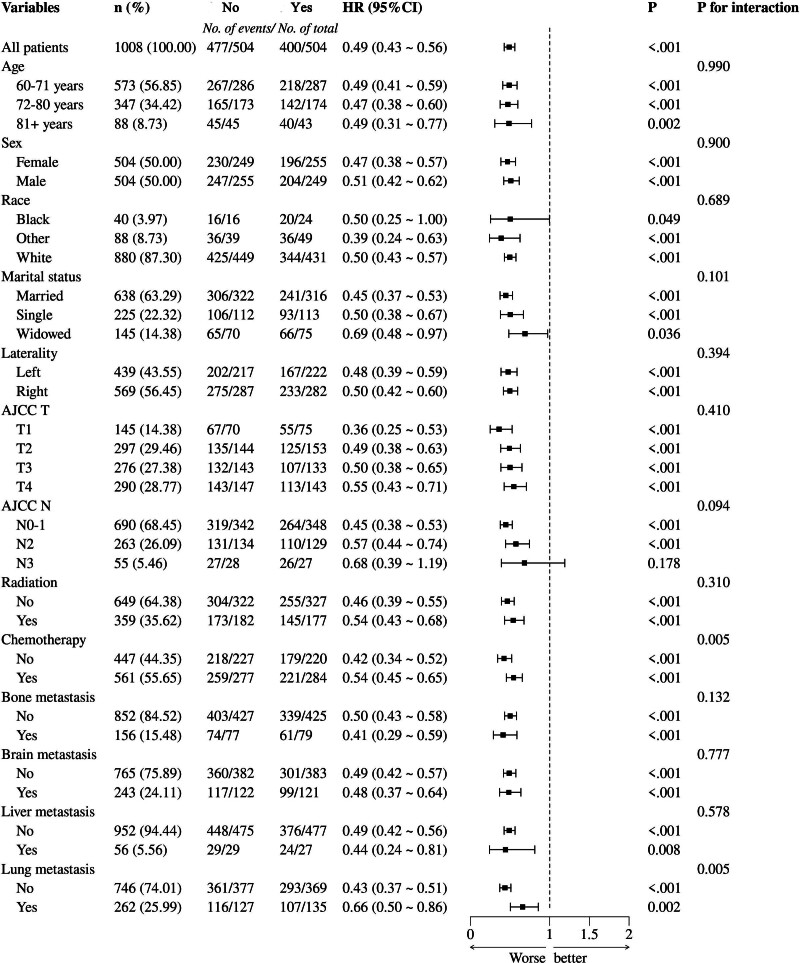
Forest maps for subgroup analysis.

Subgroup analysis revealed that the interaction between chemotherapy and lung metastasis significantly impacted the efficacy of surgical treatment in elderly patients with stage IV NSCLC (*P* <.05). Moreover, no statistically significant interaction effects were observed for other factors, such as age, gender, race, marital status, tumor location, AJCC T stage, AJCC N stage, radiotherapy, bone metastasis, brain metastasis, or liver metastasis (all *P* >.05). Surgical treatment improved patient prognosis regardless of chemotherapy administration. Surgical treatment reduced the risk of death by 58% in patients who did not receive chemotherapy (HR = 0.42, 95% CI: 0.34–0.52) and by 46% in those who received chemotherapy (HR = 0.54, 95% CI: 0.45–0.65). In the lung metastasis subgroup, surgical treatment lowered the risk of death by 57% in patients without lung metastasis (HR = 0.43, 95% CI: 0.37–0.51) and by 34% in those with lung metastasis (HR = 0.66, 95% CI: 0.50–0.86). Patients who did not receive chemotherapy and those without lung metastasis derived greater benefits from surgical treatment (Fig. 4).

## 4. Discussion

This study conducted a retrospective analysis of patients aged 60 and older with stage IV NSCLC from the SEER database, finding that surgical treatment may positively impact the prognosis of this patient group. Data analysis after propensity score matching (PSM) showed that the median OS for the surgical group was 21 months, compared to 7 months for the non-surgical group (*P* <.001). Surgical treatment reduced the risk of death by 54% (HR = 0.46, 95% CI: 0.40–0.52, *P* <.001). Subgroup analysis further indicated that patients who did not receive chemotherapy and those without lung metastasis had a significantly reduced risk of death, with reductions of 58% (HR = 0.42, 95% CI: 0.34–0.52) and 57% (HR = 0.43, 95% CI: 0.37–0.51), respectively. These results provide a reference for determining whether elderly stage IV NSCLC patients can benefit from surgical treatment, suggesting that surgery may be one of the potential treatment options for this specific patient group.

The latest cancer treatment and survival statistics indicate that elderly patients with stage IV NSCLC constitute approximately 30% of all NSCLC cases. This represents a substantial subgroup within the NSCLC population.^[9]^ Elderly patients often have multiple comorbidities and reduced visceral function, leading to their underrepresentation in prospective clinical trials. Between 1990 and 2012, elderly patients comprised only 25% of participants in clinical trials on NSCLC treatment.^[17]^ As a result, whether elderly patients with stage IV NSCLC derive benefits from surgical treatment has been predominantly investigated through retrospective studies and meta-analyses. However, these studies were not explicitly designed to focus on elderly patients, raising concerns regarding their representativeness. In a previous study, Chi-Fu Jeffrey Yang et al^[18]^ analyzed National Cancer Database data (2004–2013) on stage IV NSCLC patients undergoing surgical treatment. The final results showed that the five-year survival rate for these patients was significantly higher than for those who did not undergo surgery, at 21.1% and 5.8%, respectively. Subgroup analysis revealed that survival outcomes correlated with the local extent of the primary tumor and the scope of surgical resection. The five-year survival rates in their study closely matched our results (surgery group: 20.0%, non-surgery group: 4.0%). Although stage IV patients present with distant metastases, surgical treatment of the primary tumor has been shown to prolong OS. We speculate that this may be due to the removal of the primary lesion, which reduces tumor burden, improves local control, and ultimately extends patient survival.^[8]^ In addition, surgery can serve as a supplementary treatment to systemic therapies – such as chemotherapy, targeted therapy, or immunotherapy – when they are effective, further lowering the likelihood of recurrence. Previous retrospective studies support this observation. Compared with radiotherapy combined with chemotherapy, surgery combined with chemotherapy has been shown to extend the median survival time for stage IV NSCLC patients to approximately 20 months.^[19]^ However, the proportion of elderly patients in the aforementioned studies was below 40%, and these studies did not specifically target the elderly population. Additionally, in the subgroup analysis conducted by Chi-Fu Jeffrey Yang et al, their results differed from ours. In our study, no significant interaction *P*-value differences were detected across various T stages and N stages (*P* >.05). We speculate that this discrepancy arises from differences in N staging groupings between our study and the aforementioned research. In our study, N staging was divided into N0-1, N2, and N3, which contrasts with the groupings applied in earlier studies. Compared with N0 patients, N1 patients exhibit tumor spread to lymph nodes proximal to the primary lesion, reflecting a more aggressive tumor phenotype with a higher likelihood of lymphatic dissemination. Lymph node metastasis significantly increases the risk of disease recurrence, particularly following surgery. Therefore, the survival prognosis for N1 patients is poorer than that for N0 patients.^[20,21]^ We considered that excessive stratification of N staging might result in multicollinearity, complicating the accurate evaluation of its impact on survival outcomes. To address multicollinearity, we combined N0 and N1 patients into a single group. However, this approach might have influenced the study results, potentially explaining the discrepancies observed between our findings and those of prior research.

Chiaki Endo^[11]^ and colleagues conducted a prospective study investigating the role of surgical resection in patients with stage IV NSCLC. The study’s inclusion criteria required patients to have single-organ metastasis and undergo surgical resection of both the primary tumor and the metastatic site. The final results indicated that the 5-year survival rate among patients who underwent complete resection reached approximately 40%. This is an exceptionally high survival rate, far surpassing the findings of our study as well as all previously published data. What factors contributed to the exceptionally high survival rates reported in this study? We believe that the patients included in this study are patients primarily presenting with single-organ metastasis, including metachronous cases, rather than those initially diagnosed as Stage IV at the time of diagnosis. In contrast, all patients in our cohort were diagnosed as Stage IV at their initial presentation. Additionally, our cohort comprised patients with both single-organ and multiple-organ metastases. It is important to note that metastases involving different organs may result in distinct survival prognoses. Data from the Swedish National Cancer Registry also support this point. Compared to patients with lung metastasis and brain metastasis, patients with liver metastasis and bone metastasis have worse survival outcomes, especially for liver metastasis, where the median survival is merely 3 months. Moreover, patients with metastasis involving 2 or more organs have even worse prognoses.^[22]^ In our study, the proportion of patients with single-organ metastasis was 55%, while approximately 45% of patients had metastases involving 2 or more organs, which likely contributed to their overall poorer prognosis. Subgroup analysis from our study further supports this observation: the analysis showed that in patients without lung metastasis, surgical treatment reduced the risk of death by 57% (HR = 0.43, 95% CI: 0.37–0.51). In contrast, for patients with lung metastasis, surgical treatment only reduced the risk of death by 34% (HR = 0.66, 95% CI: 0.50–0.86). This demonstrates that fewer organ metastases are associated with a better prognosis.

Another multicenter, randomized, phase II study showed that in Stage IV NSCLC patients with progression-free disease after first-line systemic therapy, local treatments such as radiotherapy or surgery provided significant benefits in both progression-free survival and OS. Compared to patients who did not undergo surgical treatment, progression-free survival was extended by nearly 10 months, and OS was prolonged by approximately 24 months.^[23]^ Through this study, it is evident that in Stage IV NSCLC patients, the timing of intervention with chemotherapy and surgical treatment is crucial. Only when systemic disease is well-controlled by chemotherapy should local treatments such as surgery be considered. This approach allows patients to derive the maximal therapeutic benefit. Our study’s subgroup analysis also showed that the combination of surgery and chemotherapy reduced the risk of death by 46% (HR = 0.54, 95% CI: 0.45–0.65), further supporting this point. However, we also observed that for patients who did not receive chemotherapy, surgery alone reduced the risk of death by 58% (HR = 0.42, 95% CI: 0.34–0.52), a greater benefit compared to patients who received chemotherapy. In the aforementioned study, the included patients were not all elderly, whereas in our study, all patients were over 60 years old. Although chemotherapy combined with surgery can improve OS in elderly patients, it is associated with a higher incidence of treatment-related adverse events. Grade 3/4 adverse events can significantly increase the risk of mortality. We believe this is one of the reasons why patients who did not receive chemotherapy in our study exhibited greater survival benefits. However, this does not negate the role of systemic treatments such as chemotherapy. It highlights the need for careful evaluation of the optimal timing for chemotherapy and surgical interventions in the elderly population. Additionally, it is essential to identify patient subgroups who are likely to benefit most from combined chemotherapy and surgery, ultimately achieving personalized, precision-based treatment strategies.

We are the first study to explore the potential benefits of surgical treatment in elderly Stage IV NSCLC patients. Additionally, through subgroup analysis, we identified specific patient subgroups that demonstrated significant benefit. Furthermore, we employed the PSM method, allowing our results to closely approximate the effect estimates observed in randomized controlled trials. However, our study still has some limitations: first, the SEER database does not provide specific details regarding the type of surgical treatment, which limits our ability to perform more detailed analyses. Previous studies have shown that lobectomy may offer better local control and survival benefits. Therefore, we are unable to precisely evaluate the effect of this factor on survival outcomes.^[19]^ Second, with the development of therapeutic agents for lung cancer, contemporary Stage IV NSCLC patients often receive advanced systemic treatments such as ICIs and molecular targeted therapies, rather than chemotherapy alone. The changes in systemic treatment approaches could substantially influence the survival benefits associated with surgical treatment for this subset of patients. Unfortunately, the SEER database does not include information on ICIs or molecular targeted therapies.^[24,25]^ Finally, all the data in our study were derived from the SEER database, which may introduce selection bias. Therefore, future multicenter, randomized prospective studies are warranted to further confirm that elderly Stage IV NSCLC patients can benefit from surgical treatment.

## 5. Conclusion

This study demonstrates that elderly Stage IV NSCLC patients can achieve improvements in OS and reductions in mortality risk through surgical treatment. Notably, patients in the chemotherapy group and those without lung metastasis exhibited greater survival benefits.

## Acknowledgments

We gratefully acknowledge the SEER public databases for providing the data platform, the data contributors, and the developers of the R software and related packages used for retrospective analysis in this study. We also extend our thanks to all those who have contributed to scientific research.

## Author contributions

**Conceptualization:** Fang Li, Feiyang Li.

**Data curation:** Fang Li.

**Formal analysis:** Fang Li.

**Funding acquisition:** Fang Li.

**Methodology:** Fang Li, Feiyang Li.

**Project administration:** Feiyang Li.

**Resources:** Feiyang Li.

**Software:** Fang Li, Feiyang Li.

**Supervision:** Fang Li, Feiyang Li.

**Validation:** Fang Li, Feiyang Li.

**Visualization:** Feiyang Li.

**Writing – original draft:** Fang Li, Feiyang Li.

**Writing – review & editing:** Feiyang Li.
